# Evaluation of the Bioaccessibility of Essential and Toxic Trace Elements in Basil, Peppermint, and Rosemary Using an In Vitro Gastrointestinal Digestion Model

**DOI:** 10.1021/acs.jafc.4c10940

**Published:** 2025-02-28

**Authors:** Sylwia Sajkowska, Justyna Moskwa, Katarzyna Socha, Barbara Leśniewska

**Affiliations:** † Doctoral School of the University of Bialystok, Ciołkowskiego 1K, Bialystok 15-245, Poland; ‡ Department of Analytical and Inorganic Chemistry, Faculty of Chemistry, University of Bialystok, Ciołkowskiego 1K, Bialystok 15-245, Poland; § Department of Bromatology, Faculty of Pharmacy with Division of Laboratory Medicine, Medical University of Bialystok, Mickiewicza 2D, Bialystok 15-222, Poland

**Keywords:** essential elements, spice plants, enzymatic digestion, artificial dialysis membrane, lyophilization, total phenolic content, ICP–MS

## Abstract

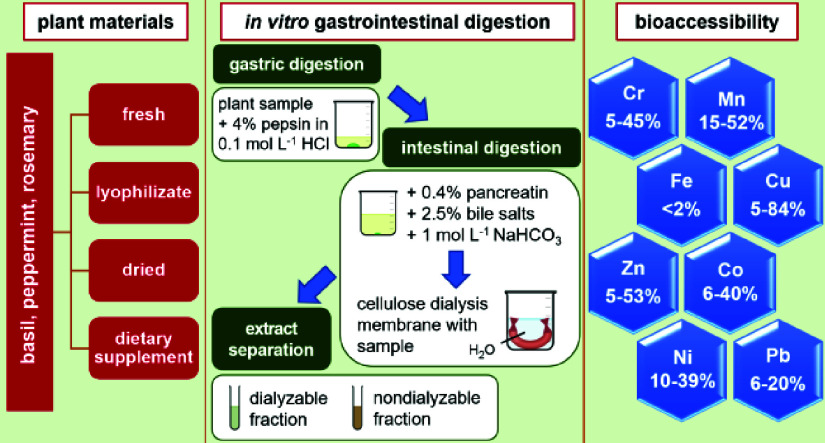

Herbs, well-known for their antioxidant properties, are a common component of the human diet. In this work, for the first time, the bioaccessibility of essential (Mn, Fe, Cu, Zn, Co, Cr, and Se) and toxic trace elements (Ni, Cd, As, Pb, and Hg) in spice plants: basil, peppermint, and rosemary was studied using an in vitro gastrointestinal digestion process and artificial dialysis membranes. The different forms of plants, fresh, lyophilized, and dried (as spice and dietary supplements), were analyzed. The results show that the bioaccessibility of elements depends on the type of plants, their form, and origin. Relatively high bioaccessibility of Cu (24–84%), Mn (39–52%), and Zn (8–43%) was observed in fresh and lyophilized herbs. The lowest value was obtained for Fe (<2%) in all herbs. The lyophilization process did not affect the trace elements’ bioaccessibility in herbs. The total phenolic content was positively correlated with the total content of elements in all tested spice plants.

## 1 Introduction

Nowadays, we cannot imagine meals without proper seasoning. Herbs have been a common component of the human diet for more than 2000 years,^1^ and they not only give aroma and flavor to our dishes but also are an excellent source of minerals, antioxidants, vitamins, and proteins.^2,3^ Therefore, to maintain a healthy diet, in addition to fruits and vegetables, it is vital to consume herbs rich in bioactive substances that have various beneficial effects on human health, including digestive stimulants, antioxidants, anti-inflammatory, antimicrobial, and anticancer effects.^4,5^ For the health benefits of herbs linked to their antioxidative activity, they are also used in pharmacological products, traditional Chinese medicine, dietary supplements, ingredients in food and beverages, or phytocosmetics.^1,6,7^ Herbal medicines, decoctions, and dietary supplements appear to be incredibly widespread and increased among patients with chronic health conditions.^6^ In the case of herbal supplements, they can be increasingly found as capsules containing dried or lyophilized herbs or their extracts, making their use much easier without the need to prepare, e.g., infusions and decoctions.

The human body requires nutrients, such as proteins, lipids, and carbohydrates, supplied in the daily diet for living and proper functioning. We also need vitamins and essential elements that perform various functions, such as building our body, regulating metabolism, and being part of enzymes (Zn, Cu, and Mn), hormones (I), and vitamins (Co). Essential trace elements have a key role in the formation of erythrocyte cells (Co, I, and Fe), regulation of glucose levels (Cr), and protection via the activation of antioxidant enzymes (Mn). They are also involved in the immune (Cu, Se, and Zn) system and the proper functioning of the brain (Cr and Mn). For example, Fe is indispensable for the synthesis of hemoglobin and myoglobin. Zn is closely related to protein synthesis and enzyme activity in the human body. In addition, Cu and Mn are vital to developing connective tissues and glucose metabolism.^8^ Although Cu and Se are essential elements for the proper functioning of the human body, if their levels change, these elements can become toxic.^9^ When Cu accumulates excessively inside cells, it can be toxic and contribute to the development of apoptotic processes, reactive oxygen species, and various diseases, such as cancer and neurological disorders. People who are chronically exposed to Se suffer from selenosis, which is characterized by abnormal nervous system functioning, skin rashes, hair loss, and garlic breath. The synthesis of thyroid and growth hormones, an insulin-like growth factor metabolism, and a disturbance of endocrine function are additional related toxic effects.^9^ The demand for essential trace elements varies greatly, and their excess or deficiency is unfavorable for the body. Despite many attempts to overcome malnutrition, deficiencies of trace elements such as Fe, Zn, I, and Se still occur in populations worldwide.^8^

The primary source of minerals is food of plant origin, but the high content of elements in such products does not always mean they benefit human health.^9^ To understand the relationship between the content of nutrients, minerals, or contaminants in food and their effects on the body, the terms bioaccessibility, bioavailability, and bioactivity were introduced. The amount or fraction released from the food matrix in the gastrointestinal tract and made available for absorption is known as bioaccessibility. Bioavailability is defined as the fraction of the amount of food ingested that reaches the organs and tissues and participates in basic metabolic processes or other biological processes. Bioactivity compasses the ability of a compound to exhibit a biological effect.^10−12^ The concept of bioaccessibility includes food’s digestive processes, which turn it into material that can be absorbed, the absorption of nutrients into intestinal epithelial cells, and, finally, presystemic metabolism, which includes both intestinal and hepatic metabolism. Bioaccessible content is always equal to or higher than bioavailable content and may be considered a proxy for the latter.^11,12^

Bioaccessibility is tested using in vitro or in vivo methods. Bioavailability is mainly determined by in vivo methods in animal or human studies, but in vitro methods are also applied.^11^ Bioactivity may be tested using ex vivo (research is conducted with organs and tissues in laboratory conditions), in vitro, and in vivo methods.^11−13^ Due to the great complexity of the gastrointestinal tract, in vitro bioaccessibility studies do not replace in vivo tests. Still, they are important for studying the effects of food digestion and the impact of the food type on the systemic bioavailability of compounds. Determining the bioaccessibility of substances in food is essential to assess their bioactivity because only ingredients released from this matrix and/or assimilated in the small intestine can become bioavailable to exert a beneficial effect on the body.^12,14^

Understanding the bioaccessibility of the elements in the tested food product is possible after appropriate modeling of the chemical, biological, and mechanical conditions prevailing in the mouth, stomach, small intestine, and large intestine, i.e., in the four sections of the digestive tract.^12^ Therefore, various approaches to in vitro methods are proposed, which involve simulating the processes occurring in the digestive tract and sections of the intestines using artificial membranes or intestinal cell lines, mainly human Caco-2 cells.^10−12^ In the literature, research about bioaccessibility is most often carried out using a two-stage simulation of the digestion process, which considers gastric and intestinal digestion.^15^ The food breakdown during the gastric digestion step is achieved by adding pepsin with the acidification of the samples to pH 2. The acidic conditions of the gastric phase cause the breakdown of most macromolecules, including proteins and carbohydrates. Samples must be acidified to pH 2 since pepsin denatures and loses activity at pH > 5. Before beginning intestinal digestion, samples must be neutralized to pH 5.5, pancreatin and bile salts must be added, and then readjusted to pH 7. Introducing pancreatic and bile enzymes facilitates the emulsification of lipids into micelles. Only lipids that are integrated into micelles may be absorbed by intestinal cells. In some cases, Caco-2 cell uptake is the next step in the simulated digestion process.^10−12,15,16^

There is also a standardized procedure for in vitro digestion testing models, such as INFOGEST.^17,18^ This is a static digestion method that maintains a constant pH for every stage of digestion and consistent meal ratios to digestive fluids. Because of this, the process is straightforward but unsuitable for modeling the kinetics of digestion. Food samples undergo sequential oral, gastric, and intestinal digestion in this method, and the determination of digestion products (e.g., peptides and amino acids, fatty acids, and simple sugars) allows for the assessment of the release of nutrients and microelements from the food matrix. In the dialyzability model, the use of various types of dialysis membranes (e.g., dialysis bag and dialysis tube) to separate the digested fraction from the residue allows for determining a dialyzable fraction containing low-molecular-weight solutes that may be bioaccessible.^17,18^

It is crucial to remember that many factors, including age, gender, health, the physiological state of the gastrointestinal system, and the kind of diet, have a considerable impact on bioavailability and can only be fully evaluated through clinical investigations.^13^ In the case of the in vitro methods, which were designed to simulate gastrointestinal functions, other parameters such as temperature, mixing dynamics, enzymatic activity, or pH levels are also important. This method has several advantages over human trials, including reduced costs, faster results, and no ethical constraints.^11,12,15−17,19^ Therefore, it was used to forecast the gastrointestinal behavior of some food components and to evaluate the bioaccessibility of elements^16,20−22^ or bioactive compounds.^23−27^

Our work aimed to study the bioaccessibility of essential (Mn, Fe, Cu, Zn, Co, Cr, and Se) and toxic trace elements (Ni, Cd, As, Pb, and Hg) in spice plants. For our research, we selected basil (*Ocimum basilicum* L.), peppermint (*Mentha × piperita* L.), and rosemary (*Rosmarinus officinalis* L.), which are often used as spices but can also be consumed as separate dishes, such as pesto or as dietary supplements. To reflect the actual way of herb consumption, fresh plants, spices (dried herbs), and dietary supplements containing capsules filled with dried plants were used as samples. Additionally, fresh plants were lyophilized to assess this herb preparation method’s impact on the bioaccessibility of elements. The tested samples were characterized by the total content of polyphenols, which are present in significant amounts in herbs and may limit the release of essential elements and their absorption in the human digestive system. To assess the bioaccessibility of essential trace elements, we applied an in vitro gastrointestinal digestion method based on mimicking the processes occurring in the stomach and small intestine using gastric and intestinal enzymes. Additionally, we used an artificial dialysis membrane to simulate intestinal absorption directly during digestion, which allowed us to simultaneously separate the dialyzable fraction of elements from the undigested residue of herbs. The inductively coupled plasma mass spectrometry (ICP–MS) method was validated to obtain good-quality results on the content of essential and toxic trace elements in herbs and their fractions after enzymatic digestion. To the best of our knowledge, this is the first study on the bioaccessibility of essential and toxic trace elements in various forms of herbs using the in vitro method.

## 2 Materials and Methods

### 2.1 Reagents

Nitric acid (69%, TraceSelect, Fluka, Germany) and hydrogen peroxide (30%, Supelco, Sigma-Aldrich, Saint Louis, USA) were used for mineralization of the herbs and nondialyzable fractions of herbs. A solid sodium bicarbonate was supplied by Panreac AppliChem (Darmstadt, Germany). Hydrochloric acid, with a 36.5 to 38% concentration, was sourced from J.T. Baker Instra-Analyzed (Phillipsburg, NJ, USA). Both reagents at appropriate concentrations (0.1 mol L^–1^ HCl, 1 mol L^–1^ NaHCO_3_, and 0.1 mol L^–1^ NaHCO_3_) were used to adjust the pH of the samples. For gastrointestinal digestion, pepsin from porcine gastric mucosa (≥500 units mg^–1^ of protein), pancreatin from the porcine pancreas (3 × USP), and bile salts were provided by Sigma-Aldrich (Steinheim, Germany). Freshly prepared solutions of both digestive enzymes were used in the experiments. Methanol used for phenolic compound extraction was obtained from Honeywell (Steinheim, Germany). Gallic acid (Sigma-Aldrich, Steinheim, Germany), Folin–Ciocâlteu’s phenol reagent (Sigma-Aldrich, Steinheim, Germany), and sodium carbonate (Chempur, Piekary Śląskie, Poland) were used for the Folin-Ciocâlteu method. The ICP multielement standard solution VIII (100 mg L^–1^ of 24 elements in 2% HNO_3_, CertiPUR, Merck, Darmstadt, Germany) was used for the preparation of working standard solutions of studied analytes. A single-element stock solution of As (Merck, Darmstadt, Germany) and Hg (Merck, Darmstadt, Germany) at the concentration of 1000 mg L^–1^ was used to prepare calibration standards. Indium standard for ICP (1000 mg L^–1^ in 2% HNO_3_, TraceCERT, Sigma-Aldrich, Steinheim, Germany) was used to prepare an internal standard solution at a concentration of 100 ng mL^–1^. Ultrapure water (18.2 MΩ cm^–1^) was obtained from a Milli-Q water purification system (Direct-Q3; Merck Millipore, Germany).

### 2.2 Instrumentation

Plant lyophilizates were gained using a freeze-dryer Alpha 1–2 LDplus (Martin Christ, Osterode am Harz, Germany). During enzymatic extraction, samples were incubated in a thermostatic shaking water bath SWB 22N (LaboPlay, Bytom, Poland). The pH values of solutions were controlled with a hand-held pH meter pH MP103 (Chemland, Stargard, Poland), equipped with an electrode SenTix. A microwave digestion system (ETHOS LEAN Compact Microwave Digestion, MILESTONE SRL, Sorisole, Italy) was used to mineralize herbs and nondialyzable fractions gained after enzymatic digestion. The UV–vis spectra of gallic acid were recorded by a UV/vis spectrophotometer (U-1900, Hitachi, Tokyo, Japan).

Determination of analytes (Cr, Mn, Fe, Co, Ni, Cu, Zn, As, Se, Cd, Hg, and Pb) in obtained solutions (after total mineralization and enzymatic digestion of herbs) was performed by ICP–MS. For this purpose, the triple quadrupole ICP–MS spectrometer (8800 ICP-QQQ, Agilent Technologies, Singapore) fitted with a MicroMist nebulizer, a Scott-type double pass spray chamber Peltier cooled, Ni sampler and Pt skimmer cones, and a collision/reaction cell octopole reaction system (ORS^3^) was used. Samples were introduced directly into ICP–MS using an SPS 4 autosampler. Helium as a collision gas (for Fe, Co, Ni, Cd, Hg, and Pb), ammonia (for Cr, Mn, Cu, and Zn), and oxygen (for As and Se) as reaction gases in ORS^3^ were used for the elimination of the polyatomic interferences during the determination of analytes. The optimized operating conditions are listed in Table 1. The data was processed using Agilent Mass Hunter software.

**Table 1:** Operating Conditions Used for Determination of Cr, Mn, Fe, Co, Ni, Cu, Zn, As, Se, Cd, Hg, and Pb by the ICP–MS Method (8800 ICP-QQQ)

parameter	value
RF power	1550 W
plasma gas flow rate, auxiliary gas flow rate, nebulizer gas flow rate	15 L min^–1^, 0.9 L min^–1^, 1.08 L min^–1^
spray chamber temperature	2 °C
sample depth	10 mm
ORS^3^ gas flow rate and energy discrimination	He: 4 mL min^–1^; NH_3_: 2 mL min^–1^; O_2_: 0.4 mL min^–1^; He: 5 V; NH_3_: −7 V; O_2_: −7 V
scan type	MS/MS
replicates	3
sweeps/replicate	100
integration time	0.1 s (Mn, Fe, Co, Cd); 0.3 s (Cr, Ni, Cu, Zn, Pb); 1.0 s (As, Se, Hg)
monitored ion (*m*/*z*)	^52^Cr, ^55^Mn, ^56^Fe, ^59^Co, ^60^Ni, ^65^Cu, ^66^Zn, ^75^As^16^O, ^78^Se^16^O, ^111^Cd, ^201^Hg, ^208^Pb
internal standard (*m*/*z*)	^115^In

### 2.3 Plant Materials

The fresh herbs of basil (*O. basilicum* L.), peppermint (*Mentha × piperita* L.), and rosemary (*R. officinalis* L.) (two plants of each herb) were purchased from a gardener who cultivates plants for sale in Poland. The dried basil, peppermint, and rosemary plant leaves (25 g of each herb) from organic cultivation were obtained from commercial sources. The tested supplements (packing 120 capsules) containing 400 mg of dried ground plant leaves in their capsules were also purchased from a commercial source. Part of the fresh basil, peppermint, and rosemary leaves (three samples of 10 g each of the herbs) were lyophilized using a freeze-dryer. Based on the difference in mass samples before and after lyophilization, the water content in herbs was evaluated as follows: 89.6% in basil, 85.1% in peppermint, and 74.4% in rosemary. The obtained masses of lyophilized herbs were in the range of 3 g (basil) to 6 g (rosemary).

Certified reference material (CRM) of Mixed Polish Herbs INCT-MPH-2 with a certified content of 35 elements, produced by the Department of Analytical Chemistry, Institute of Nuclear Chemistry and Technology, Warsaw, Poland, was used for quality control of measurements by the ICP–MS method.

### 2.4 In Vitro Digestion Procedure

**Figure 1:**
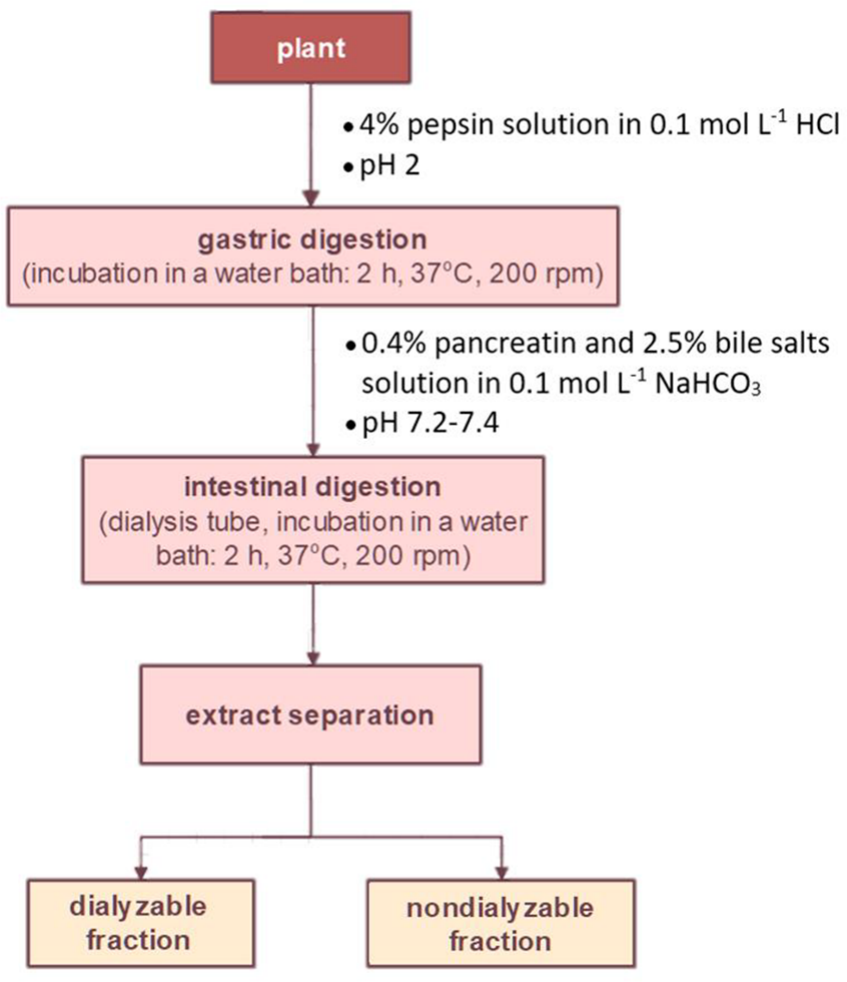
Analytical procedure for plants’ in vitro gastrointestinal digestion.

An in vitro digestion procedure using an artificial membrane was applied to study the bioaccessibility of essential and toxic metals in herbs (Figure 1). Fresh herb leaves, approximately 10 g each were collected directly before the in vitro digestion procedure. Fresh, dried, and lyophilized herbs were thoroughly mixed before sampling. The contents of 20 capsules of the dietary supplement were placed in one container and also thoroughly mixed to obtain a representative sample. Next, the plant leaves: 2.5 g of fresh/dried/dietary supplements or 0.5 g of lyophilized plants (corresponding to 2.5 g of fresh plants) was weighed into polyethylene vessels with a volume of 100 mL. Then, 15 mL of a 4% pepsin solution in 0.1 mol L^–1^ HCl was added, and the obtained mixtures (pH = 2) were incubated for 2 h at 37 °C in a thermostatic shaking water bath at 200 rpm. After this time, the pH of the samples was adjusted to a value of 7.2–7.4 (similar to the pH in the intestinal tract) by using 1 mol L^–1^ NaHCO_3_. Then, 5 mL of 0.4% pancreatin and 2.5% bile salt solution in 0.1 mol L^–1^ NaHCO_3_ was added to each sample. Then, the entire content of the vessels was quantitatively transferred to the dialysis tubing cellulose membranes (14 kDa molecular weight cutoff, Sigma-Aldrich, Steinheim, Germany), which were earlier prepared (treated with 0.1 mol L^–1^ HCl for 24 h and washed out with Milli-Q water). Properly protected dialysis tubing cellulose membranes were placed into polyethylene vessels containing 60 mL of Milli-Q water and incubated in a thermostatic shaking water bath for 2 h at 37 °C at 200 rpm. After enzymatic digestion, two fractions of herbs were obtained: nondialyzable (the remaining content in the tubing) and dialyzable (the solution outside the tubing). Samples and control samples were prepared in triplicate. Samples were stored at −12 °C until analysis.

### 2.5 Sample Preparation for the Determination of Essential and Toxic Trace Elements by the ICP–MS Method

#### 2.5.1 Microwave-Assisted Acid Digestion of Herbs and Nondialyzable Fractions of Herbs

In order to determine the total content of the analytes in the tested herbs samples, microwave-assisted digestion was carried out. The sample preparation procedure was as follows: 0.1 g of the sample was accurately weighed into a polytetrafluoroethylene vessel; next, 3.0 mL of concentrated HNO_3_ and 0.5 mL of 30%H_2_O_2_ were added. The heating program consisted of three stages: 10 min at a power of 900 W and a temperature of up to 160 °C, 10 min at a power of 900 W and a temperature of up to 200 °C, and then 15 min at a power of 900 W and a temperature of 200 °C. After digestion, the obtained solutions were diluted with Milli-Q water and analyzed using the ICP–MS method. The same procedure was applied for the acid digestion of the CRM of INCT-MPH-2 and nondialyzable fractions of herbs. Samples and reagent blanks were obtained in triplicate.

#### 2.5.2 Digestion of Dialyzable Fractions of Herbs

The samples of dialyzable fractions were treated with concentrated nitric acid to determine the metal content. To the 250 μL of dialyzable fractions, a small volume of concentrated HNO_3_ was added, samples were left for about 20 min for mineralization of the organic matrix, and then Milli-Q water was added to obtain the final concentration of HNO_3_ in samples equal to 2%. All samples and reagent blanks were prepared in triplicate and analyzed using the ICP–MS method.

### 2.6 Determination of Cr, Mn, Fe, Co, Ni, Cu, Zn, Se, Cd, Pb, As, and Hg by the ICP–MS Method

The ICP multielement standard solution VIII at the concentration of 100 mg L^–1^ and single-element stock solutions of As and Hg were used to prepare the Cr, Mn, Fe, Co, Ni, Cu, Zn, Se, Cd, Pb, As, and Hg calibration curves. Appropriate dilutions made it possible to obtain solutions for calibration curves of the analytes with the concentration range 0.25–100 ng mL^–1^ for Cr, Co, Ni, Cu, Zn, Cd, Pb, As, Se, and Hg and 1–500 ng mL^–1^ for Mn and Fe in 2% HNO_3_. The blank sample was a 2% nitric acid solution. The solution of In at the concentration of 100 ng mL^–1^ was used as an internal standard to compensate for any signal changes due to the matrix’s presence. All measurements of the signal intensity of analytes by the ICP–MS method were performed according to the instrumental conditions given in Table 1. All samples of herbs after total digestion and in vitro gastrointestinal digestion were diluted correctly and analyzed using the ICP–MS method.

Quality control of the ICP–MS method was performed using the CRM of INCT-MPH-2. The determined contents of the studied elements in CRM INCT-MPH-2 were compared with the certified values. The declared concentrations for tested elements in the CRM were as follows (certified value ± U, *k* = 2): 1.69 ± 0.13 μg g^–1^ for Cr, 191 ± 12 μg g^–1^ for Mn, 460 μg g^–1^ for Fe, 210 ± 25 ng g^–1^ for Co, 1.57 ± 0.16 μg g^–1^ for Ni, 7.77 ± 0.53 μg g^–1^ for Cu, 33.5 ± 2.1 μg g^–1^ for Zn, 191 ± 23 ng g^–1^ for As, 199 ± 15 ng g^–1^ for Cd, 17.6 ± 1.6 ng g^–1^ for Hg, and 2.16 ± 0.23 μg g^–1^ for Pb.

### 2.7 Determination of Total Phenolic Content in Herbs

#### 2.7.1 Extraction of Phenolic Compounds from Herbs

To prepare samples for the determination of total phenolic content (TPC) in herbs, the extraction was performed according to the method reported by Pereira et al.^28^ with slight modifications. To the centrifuge tubes, 50 mg of fresh/lyophilized/dried/dietary supplement plant leaves was weighed, and 5 mL of the extraction solution containing 50% (v/v) methanol and 1.2 mol L^–1^ HCl was added. Then, the samples were shaken on the vortex for 1 min and placed in a water bath at 90 °C for 3 h. After this time, the samples were cooled to room temperature and centrifuged at 4000 rpm for 20 min. The obtained supernatants were collected for further steps.

#### 2.7.2 Determination of TPC by the Folin-Ciocâlteu Method

The Folin-Ciocâlteu method was used for the determination of the TPC in herbs. First, a series of standard solutions of gallic acid were prepared in the concentration range from 20 to 300 μg mL^–1^. Then, 100 μL of individual standard solutions, 500 μL of Folin-Ciocâlteu reagent, and 1000 μL of Milli-Q water were taken and mixed. After 1 min, 1500 μL of a 20% Na_2_CO_3_ solution was added and mixed again. Similarly, samples were prepared by taking 100 μL of obtained supernatants in the previous step instead of the gallic acid solution. In the case of supernatants from the herbs in the form of lyophilized, dried, and dietary supplements, samples were diluted 10 times before using the Folin-Ciocâlteu reagent. The standards and samples were left in a dark place for 30 min. Then, the absorbances of the prepared standard solutions and samples were measured at a wavelength of 752 nm. The results were expressed as milligrams of gallic acid per 1 g of herb.

### 2.8 Method for Evaluation of Bioaccessibility of Essential and Toxic Trace Elements in Herbs

The bioaccessibility ratios of the elements (Cr, Mn, Fe, Cu, Zn, Co, Ni, and Pb), expressed as a percentage, for the in vitro digestion model were calculated using the following equation^16^
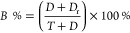
where *B* % is the percentage of the bioaccessibility (relative bioaccessibility) of studied elements, *D* is the amount of the element (μg) in the dialyzable fraction, *D*_r_ is the amount of the element (μg) corresponding to the equilibrium of concentrations on both sides of the cellulose membrane inside the dialysis tube, and *T* is the amount of the element (μg) present in the digest of the nondialyzable fraction.

*D*_r_ was calculated using the following equation^16^
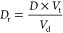
where *V*_t_ is the volume of the dialysis tube (mL) and *V*_d_ is the volume of dialyzable fraction (mL).

### 2.9 Statistical Analysis

The obtained data were elaborated and analyzed using Excel 2016 (Copyright Microsoft Excel 2016, Redmond, WA, USA). All data were presented as the mean value and standard deviation (SD, *n* = 3). Statistical comparisons between the bioaccessibility of elements in various forms of plants were performed using the Fisher-Snedecor F test and Student’s *t*-test. Pearson’s correlation was used to evaluate the correlation between TPC, bioaccessibility of elements, and total contents of elements in herbs. Heatmap discrimination between the elements’ bioaccessibility from plants was produced in Statistica 13 (StatSoft, Poland) software.

## 3 Results and Discussion

The bioaccessibility of Cr, Mn, Fe, Co, Ni, Cu, Zn, As, Se, Cd, Hg, and Pb in spice plants was evaluated by the application of an in vitro gastrointestinal digestion method using gastric and intestine enzymes and an artificial dialysis membrane. To obtain accurate and reliable results on the bioaccessibility of essential and toxic metals, the ICP–MS method was validated.

### 3.1 Validation of the ICP–MS Method for Determination of Cr, Mn, Fe, Co, Ni, Cu, Zn, As, Se, Cd, Hg, and Pb in Herbs and Their Fractions after In Vitro Gastrointestinal Digestion

To obtain good-quality results, the procedure for the determination of essential and toxic trace elements by using the ICP–MS method was validated. The subsequent parameters were evaluated: linearity, limit of detection (LOD) and limit of quantification (LOQ), precision, repeatability, trueness and uncertainty of results, and the obtained validation parameters are presented in Table 2.

**Table 2:** Validation Parameters of the ICP–MS Method for Determination of Cr, Mn, Fe, Co, Ni, Cu, Zn, As, Se, Cd, Hg, and Pb in Herbs and Their Fractions after In Vitro Gastrointestinal Digestion

analyte	*r*	LOD, ng mL^–1^	LOQ, ng mL^–1^	precision, CV, %, *n* = 6	repeatability, CV, %, *n* = 5	trueness, CRM recovery ±SD, %, *n* = 3	expanded uncertainty U, %, *k* = 2
Cr	1.0000	0.33	0.41	0.2–1.6	3.7	90 ± 3	15
Mn	1.0000	0.59	0.83	0.1–2.9	5.1	102 ± 5	8.6
Fe	1.0000	10.0	12.5	0.6–9.0	6.6	103 ± 7	
Co	0.9999	0.015	0.024	0.8–9.4	8.5	103 ± 9	14
Ni	0.9999	0.28	0.33	0.6–8.4	7.9	108 ± 8	12
Cu	1.0000	0.63	0.75	0.3–4.2	7.8	102 ± 8	9.8
Zn	1.0000	2.55	2.72	0.1–4.0	7.4	101 ± 7	11
As	1.0000	0.021	0.039	2.4–6.8	7.3	102 ± 8	13
Se	0.9999	0.027	0.046	0.9–10.6			
Cd	0.9999	0.010	0.014	0.2–9.0	8.5	100 ± 8	11
Hg	0.9977	0.023	0.042	0.3–9.4			
Pb	0.9992	1.16	1.82	0.2–8.2	9.9	101 ± 10	18

Calibration curves of analytes were obtained by measuring in triplicate solutions at seven concentration levels in the range of 0.25–100 ng mL^–1^ for Cr, Co, Ni, Cu, Zn, Cd, Pb, As, Se, and Hg, and 1–500 ng mL^–1^ for Mn and Fe. The obtained calibration curves of all analytes were characterized by a good correlation coefficient (*r* = 0.9977–1.0000) (Table 2).

In general, the LOD is defined as the lowest possible concentration that can be measured reliably, whereas the LOQ is the lowest concentration of an analyte that can be determined with an acceptable level of precision and trueness. The value of LOD was calculated based on the signals of the blank and its SD (blank ± 3 SD) substituted into the equation of the calibration curve (*y = bx + a*). The mineralized, nondialyzable fraction of the enzyme solution after the in vitro procedure, prepared as described in Section 2.5.1, was used as a blank sample. The value of LOQ was calculated using the signals of the blank, 10 times the SD of the blank, and the equation of the calibration curve. In this study, the lowest values of these parameters were obtained for Cd (LOD = 10 pg mL^–1^, LOQ = 14 pg mL^–1^) and Co (LOD = 15 pg mL^–1^, LOQ = 24 pg mL^–1^), while the highest values were obtained for Fe (LOD = 10 ng mL^–1^, LOQ = 12.5 ng mL^–1^) (Table 2).

Precision is the closeness of agreement between measured values obtained by replicating measurements of the same or similar quantities under specified stable conditions. During research, the precision of the measurements was determined as the range of coefficient of variation (CV) values from the intensity signals of standard solutions of analytes at seven concentration levels. The obtained coefficients of variation for all elements showed that the precision of the used method was satisfactory (Table 2).

Repeatability is precision under conditions that include the same measurement procedure, operators, measuring system, operation conditions, location, and replication measurements on the same or similar objects over a short time. Repeatability, expressed as the CV, was evaluated by measuring three parallel CRM samples after total mineralization over 3 weeks (5 measurement days). The results of repeatability are satisfactory for all tested elements (CV < 10%, Table 2).

The trueness of the method was evaluated by analysis of the CRM (INCT-MPH-2). The recoveries of analytes were calculated based on the content of the elements determined by the ICP–MS method and the certified reference values. The obtained values were in the range of 90% (for Cr) to 108% (for Ni) (Table 2). In order to investigate whether the obtained recoveries are significantly different from 100%, the Student’s *t*-test was performed. In all cases, the obtained values of *t*_calc_ were lower than the values of *t*_crit_ equal 2.776 at α = 0.05, proving that the ICP–MS method’s trueness is good.

The expanded uncertainty (U, %) of the content of the elements in CRM INCT-MPH-2 was evaluated based on the method validation parameters: repeatability and trueness of the method. This value was obtained by multiplying the combined standard uncertainty by a coverage factor *k* = 2, which resulted in a confidence level of approximately *p* = 95%. To calculate the combined standard uncertainty, the following formula was used
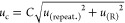
where *u*_(repeat.)_ was the standard uncertainty of repeatability calculated as a relative SD, and *u*_(R)_ was the standard uncertainty of recovery. The obtained results of expanded uncertainty were below 18% (Table 2). The greatest share of those values was taken by the standard uncertainty of recovery.

### 3.2 Total Contents of Essential and Toxic Trace Elements in Herbs

The total contents of the selected essential elements as Cr, Mn, Fe, Cu, Zn, Co, and Se and some toxic metals, such as Ni, As, Cd, Pb, and Hg in the tested herbs were determined by the ICP–MS method after total acidic digestion. Based on the results obtained for the total metal contents in various forms of herbs (Table 3), it was observed that the highest amounts of Mn and Zn were found in basil in lyophilized form, whereas Fe, Cu, and Cr were found in a dietary supplement based on basil. Fe was most abundant in a dietary supplement based on basil (1187 μg g^–1^), peppermint (449 μg g^–1^), and in dried rosemary (897 μg g^–1^). The greatest amount of Mn was in basil (345 μg g^–1^) and rosemary (81.4 μg g^–1^) in lyophilized form and peppermint as a dietary supplement (132 μg g^–1^). Cu has been found in the highest amounts in basil (16.9 μg g^–1^) and peppermint (10.7 μg g^–1^) dietary supplements and dried rosemary (6.66 μg g^–1^). Zinc was found mainly in lyophilized basil (91.6 μg g^–1^) and rosemary (43.8 μg g^–1^) and in a peppermint-based dietary supplement (45.4 μg g^–1^). The highest content of Cr (3.52 μg g^–1^) was found in a dietary supplement based on basil, whereas Co (1.79 μg g^–1^) and Se (135 ng g^–1^) were found in dried basil. Among toxic metals, Hg was not detected in any of the tested herbs. Cd content was at the level 8.8–115 ng g^–1^, As in the range of 20–1148 ng g^–1^, whereas the content of Pb was in the range of 419–1884 ng g^–1^ with the highest value in dried rosemary. The content of Ni was at the level 0.36–2.47 μg g^–1^, with the highest amount of this element in a dietary supplement based on basil.

**Table 3:** Total Content of Essential and Toxic Trace Elements Determined by the ICP–MS Method in Herbs after Total Digestion, and TPC Determined by Folin–Ciocâlteu Method in Herbs

	basil				peppermint				rosemary			
	fresh^a^	lyophilizate	dried	supplement	fresh^a^	lyophilizate	dried	supplement	fresh^a^	lyophilizate	dried	supplement
	element content ±SD, μg g^–1^, *n* = 3											
Cr	0.83 ± 0.02	1.23 ± 0.08	2.20 ± 0.03	3.52 ± 0.26	0.26 ± 0.01	0.70 ± 0.05	0.67 ± 0.06	1.31 ± 0.01	0.28 ± 0.01	0.73 ± 0.06	2.76 ± 0.03	0.77 ± 0.05
Mn	46.7 ± 0.8	345 ± 15	139 ± 3	53.3 ± 1.9	15.7 ± 0.2	105 ± 1	42.4 ± 0.6	132 ± 4	15.3 ± 0.2	81.4 ± 0.3	38.1 ± 1.3	24.5 ± 0.7
Fe	33.6 ± 0.8	319 ± 19	769 ± 13	1187 ± 55	44.9 ± 0.7	257 ± 6	222 ± 8	449 ± 1	128 ± 3	636 ± 7	897 ± 57	352 ± 6
Ni	0.71 ± 0.12	1.71 ± 0.08	1.84 ± 0.01	2.47 ± 0.23	0.76 ± 0.02	1.16 ± 0.05	0.69 ± 0.14	2.38 ± 0.04	0.36 ± 0.03	0.72 ± 0.06	1.44 ± 0.31	0.60 ± 0.02
Cu	2.04 ± 0.06	12.7 ± 0.3	8.63 ± 0.16	16.9 ± 0.7	2.11 ± 0.09	9.68 ± 0.07	4.91 ± 0.16	10.7 ± 0.4	0.65 ± 0.03	2.19 ± 0.01	6.66 ± 0.42	5.59 ± 0.17
Zn	11.4 ± 0.8	91.6 ± 4.7	19.1 ± 0.5	40.7 ± 0.7	10.0 ± 0.7	42.2 ± 1.6	30.3 ± 0.6	45.4 ± 1.4	12.4 ± 1.1	43.8 ± 0.2	24.6 ± 0.5	17.4 ± 0.5

	element content ±SD, ng g^–1^, *n* = 3											
Co	18.2 ± 0.6	58.9 ± 21.8	1788 ± 132	588 ± 35	<LOQ	82.4 ± 3.0	72.3 ± 10.9	189 ± 13	<LOQ	53.0 ± 1.0	395 ± 10	154 ± 2
As	38.6 ± 0.8	140 ± 6	1148 ± 89	376 ± 14	20.1 ± 1.7	184 ± 5	66.7 ± 4.0	135 ± 10	26.9 ± 0.8	98.0 ± 10.0	274 ± 12	136 ± 1
Cd	<LOQ	14.1 ± 0.9	29.6 ± 0.7	50.9 ± 4.3	<LOQ	43.9 ± 1.2	31.7 ± 3.2	115 ± 10	<LOQ	21.9 ± 2.9	19.0 ± 1.1	8.75 + 1.02
Pb	<LOD	472 ± 97	564 ± 35	1096 ± 65	<LOD	419 ± 61	<LOD	789 ± 30	<LOD	<LOD	1884 ± 96	506 ± 18
Se	<LOD	<LOD	135 ± 12	110 ± 6	<LOD	<LOD	32.0 ± 3.8	31.4 ± 2.1	<LOD	<LOD	56.1 ± 1.8	30.3 ± 3.2
Hg	<LOD	<LOD	<LOD	<LOD	<LOD	<LOD	<LOD	<LOD	<LOD	<LOD	<LOD	<LOD

	total phenolic content (TPC) expressed as gallic acid content ±SD, mg g^–1^, *n* = 3											
TPC	7.7 ± 1.3	64.7 ± 5.2	84.6 ± 1.0	45.1 ± 1.5	18.7 ± 2.5	86.9 ± 8.9	92.6 ± 0.6	56.3 ± 3.3	19.0 ± 2.1	66.9 ± 8.8	78.1 ± 4.0	67.1 ± 0.5

a For wet mass.

The obtained results regarding the content of Mn, Fe, Cu, and Zn in fresh and dried plants are similar to those in the publications.^1,29−31^ The content of Mn, Fe, Cu, and Zn in dried basil (Table 3) was similar to the content of Mn (98–145 μg g^–1^) and Fe (1179–1412 μg g^–1^) in Spanish herbs and the content of Cu (8–19 μg g^–1^) and Zn (16–58 μg g^–1^) in Moroccan herbs.^30^ In lyophilized basil, the high content of Mn (345 μg g^–1^) was at the level determined in Moroccan herb samples (111–387 μg g^–1^).^30^ In fresh basil, the content of Fe and Zn was similar to the values presented by Filip,^29^ i.e., 31.7 μg g^–1^ Fe and 8.1 μg g^–1^ Zn. In the case of dried mint, the Mn and Zn content (Table 3) corresponded to the values determined in mint from Italy (131 μg g^–1^ Mn and 45.9 μg g^–1^ Zn), while the Cu and Fe content was similar to those in samples from Italy (9.5 μg g^–1^ Cu and 107 μg g^–1^ Fe) and Tunisia (7.1 μg g^–1^ Cu and 330 μg g^–1^ Fe).^1^ The Mn, Fe, Cu, and Zn content in rosemary (Table 3) was similar to those in dried samples from Turkey (20–30 μg g^–1^ Mn, 500–760 μg g^–1^ Fe, 4.7–4.9 μg g^–1^ Cu, and 27–49 μg g^–1^ Zn),^31^ Tunisia (14 μg g^–1^ Zn), and Italy (5.0 μg g^–1^ Cu).^1^ The content of toxic elements, As and Pb, in dried rosemary was similar to those determined in samples from Tunisia (383 ng g^–1^ As and 1418 ng g^–1^ Pb).^1^ It is worth mentioning that the total content of elements in herbs depends on many factors, including the type of element, plant species, physical and chemical properties of the soil, soil contamination, application of natural or artificial fertilizers, cultivation conditions, and other factors. Therefore, we observed a difference in metal contents in the same type of herbs but present in lyophilized or dried form because those plants came from different locations.^1^

#### 3.2.1 Health Risk Assessment and the Coverage of Recommended Dietary Allowance for Humans

The determined contents of Cd and Pb in herbs do not exceed the maximum permissible levels of these metals in vegetables and herbs (0.1 μg g^–1^ Cd) and dietary supplements (1 μg g^–1^ Cd and 3 μg g^–1^ Pb) according to EU regulation.^32^ However, to evaluate health risks, the doses of toxic elements taken up by the human body were calculated based on the provisional tolerable monthly intake (PTMI) for Cd and the benchmark dose lower confidence limit (BMDL) for As and Pb. PTMI is the safe level of intake of a contaminant, considering both food and nonfood sources in μg kg^–1^ b.w. per month. The PTMI value for Cd is 25 μg kg^–1^ b.w., reflecting the long half-life of Cd in humans.^33^ It was found that consuming 1 g of the tested herbs daily by an adult weighing 60 kg during a month will introduce Cd into the body at a level not exceeding 0.23% PTMI (Table S1). The BMDL is the minimum dose of a substance that produces a clear, low-level health risk, usually in the range of a 1–10% change in a specific toxic effect, such as, e.g., cancer induction. The BMDL, evaluated based on 1–10% of benchmark response (BMR), is usually used as the reference point to assess the potential risks of exposure to a given hazard. BMDL arsenic of 0.06 μg kg^–1^ b.w. per day was derived from an epidemiological study showing that inorganic arsenic causes skin cancer.^34^ BMDL dietary lead intake values in adults of 1.50 μg kg^–1^ b.w. per day and 0.63 μg kg^–1^ b.w. per day were derived for the cardiovascular and kidney effects, respectively.^35^ It was found that consuming 1 g of the tested herbs daily by an adult weighing 60 kg will introduce As at a level of 0.75 to 10.4% of BDML (exceptionally 32% of BMDL in the case of dried basil) (Table S1). In the case of Pb, consumption of herbs may introduce into the body of an adult person 1.1 to 5% of BMDL concerning kidney effects or 0.5 to 2% of BMDL for cardiovascular effects (Table S1). Therefore, it may be concluded that the consumption of spice plants does not induce a significant health risk connected to toxic trace elements such as As, Cd, and Pb.

Based on the content of Mn, Fe, Cu, and Zn in tested herbs, their daily supply was calculated for adult consumers aged 31–50. This group was selected as one of the largest groups of the population, which most often consumes herbs as spices or dietary supplements and consciously chooses such products to maintain good health. The percentage of the coverage demand of adult consumers was estimated based on the recommended dietary allowance (RDA) for Poland^36^ at the following levels for women and men, respectively: Mn 1.8 and 2.3 mg, Fe 18 and 10 mg, Cu 0.9 and 0.9 mg, and Zn 8 and 11 mg. The consumption of 1 g of herbs tested in our study could cover 0.1–1.9% of RDA for Cu, 0.1–1.2% of RDA for Zn, 0.2–11.9% of RDA for Fe, and 0.7–19% of RDA for Mn (Table S2). On the other hand, consuming the herbs tested in larger quantities, e.g., in the form of pesto from fresh plants (approximately 25 g of leaves), can significantly meet the daily requirement for Mn at a level of 51–65% in the case of basil and 17–22% in the case of peppermint and rosemary. However, it covers the daily supply for Cu (1.8–5.9% of the RDA) and Zn (2.3–3.9% of the RDA) to a lesser extent (Table S2). Therefore, even a small amount of herbs in a diet can provide a good source of supplementation for mineral deficiency.

### 3.3 Mass-Balance Study

The mass-balance study was carried out to indicate whether the obtained results of metal concentrations in dialyzable and nondialyzable fractions are consistent with the determined total content of these metals in plants.^37^ For this purpose, the sum of the content of each element in both fractions was compared with its total content in herbs and expressed as agreement in %. The obtained results for the in vitro digestion method are shown in Table 4. It was observed that the obtained values of the agreement for all metals were quantitative within the following range: 85–102% for Mn, 85–112% for Fe, 86–109% for Cu, 88–110% for Zn, 81–115% for Co, 80–118% for Ni, and 83–107% for Pb. This indicates that during the complicated procedure of enzymatic digestion of the tested herbs, no significant losses of analytes or contamination of samples occurred.

**Table 4:** Mass Balance Study of In Vitro Gastrointestinal Digestion of Basil, Peppermint, and Rosemary in Various Forms (Fresh, Lyophilizate, Dried, and Dietary Supplement) for Cr, Mn, Fe, Cu, Zn, Co, Ni, and Pb Determined by ICP–MS^a^

		basil				peppermint				rosemary			
		fresh	lyophilizate	dried	supplement	fresh	lyophilizate	dried	supplement	fresh	lyophilizate	dried	supplement
		element content ±SD, μg g^–1^, *n* = 3											
Cr	total content	0.83 ± 0.02	1.23 ± 0.08	2.20 ± 0.03	3.52 ± 0.26	0.26 ± 0.01	0.70 ± 0.05	0.67 ± 0.06	1.31 ± 0.01	0.28 ± 0.01	0.73 ± 0.06	2.76 ± 0.03	0.77 ± 0.05
	dialyzable fraction	0.06 ± 0.00	0.26 ± 0.02	0.10 ± 0.01	0.09 ± 0.01	0.06 ± 0.00	0.13 ± 0.01	0.06 ± 0.00	0.05 ± 0.00	0.07 ± 0.01	0.19 ± 0.02	<LOQ	0.04 ± 0.00
	nondialyzable fraction	0.83 ± 0.02	1.10 ± 0.07	1.80 ± 0.08	3.16 ± 0.30	0.21 ± 0.01	0.63 ± 0.09	0.58 ± 0.07	1.13 ± 0.11	0.20 ± 0.01	0.54 ± 0.03	1.68 ± 0.09	0.75 ± 0.05
	sum (dialyzable + nondialyzable)	0.89 ± 0.07	1.36 ± 0.03	1.90 ± 0.06	3.25 ± 0.22	0.27 ± 0.01	0.75 ± 0.06	0.64 ± 0.05	1.18 ± 0.08	0.27 ± 0.01	0.73 ± 0.01		0.79 ± 0.05
	agreement, %	107 ± 9	111 ± 8	86.5 ± 3.8	92.3 ± 7.3	103 ± 3	108 ± 14	96.1 ± 6.5	90.7 ± 6.2	96.9 ± 8.9	100 ± 8		103 ± 11
Mn	total content	46.7 ± 0.8	345 ± 15	139 ± 3	53.3 ± 1.9	15.7 ± 0.2	105 ± 1	42.4 ± 0.6	132 ± 4	15.3 ± 0.2	81.4 ± 0.3	38.1 ± 1.3	24.5 ± 0.7
	dialyzable fraction	12.8 ± 0.9	70.6 ± 2.8	28.5 ± 1.9	4.24 ± 0.22	4.32 ± 0.13	24.4 ± 1.5	4.59 ± 0.09	9.62 ± 0.24	3.86 ± 0.06	20.6 ± 1.0	6.79 ± 0.36	3.25 ± 0.09
	nondialyzable fraction	34.7 ± 2.0	250 ± 24	103 ± 3	45.1 ± 0.4	11.5 ± 0.8	73.9 ± 5.2	31.9 ± 1.0	107 ± 1	11.1 ± 0.6	49.1 ± 4.0	25.6 ± 0.8	18.4 ± 0.4
	sum (dialyzable + nondialyzable)	47.5 ± 2.3	320 ± 26	132 ± 1	49.3 ± 0.6	15.9 ± 1.1	98.3 ± 5.5	36.5 ± 0.7	117 ± 1	15.0 ± 0.4	69.8 ± 4.7	32.4 ± 0.3	21.7 ± 0.3
	agreement, %	102 ± 6	92.8 ± 4.8	94.9 ± 2.1	92.6 ± 4.4	101 ± 6	93.7 ± 5.2	85.0 ± 2.2	88.6 ± 2.4	98.4 ± 0.8	85.8 ± 5.9	85.3 ± 2.5	88.4 ± 3.7
Fe	total content	33.6 ± 0.8	319 ± 19	769 ± 13	1187 ± 55	44.9 ± 0.7	257 ± 6	222 ± 8	449 ± 1	128 ± 3	636 ± 7	897 ± 57	352 ± 6
	dialyzable fraction	0.31 ± 0.01	2.22 ± 0.12	0.76 ± 0.01	1.49 ± 0.05	0.11 ± 0.02	1.43 ± 0.11	0.27 ± 0.02	0.28 ± 0.01	0.77 ± 0.01	3.03 ± 0.04	0.16 ± 0.01	0.14 ± 0.01
	nondialyzable fraction	29.6 ± 1.5	292 ± 8	679 ± 32	1152 ± 42	45.0 ± 4.2	285 ± 13	192 ± 11	404 ± 2	108 ± 2	605 ± 19	760 ± 25	332 ± 7
	sum (dialyzable + nondialyzable)	29.9 ± 1.0	294 ± 8	680 ± 9	1154 ± 42	45.1 ± 4.2	287 ± 9	192 ± 8	404 ± 1	109 ± 1	608 ± 14	761 ± 18	333 ± 5
	agreement, %	89.0 ± 4.6	92.2 ± 3.2	88.4 ± 2.6	97.3 ± 4.6	100 ± 8	112 ± 2	86.6 ± 5.8	90.0 ± 0.4	85.0 ± 3.1	95.5 ± 1.4	85.1 ± 7.0	94.6 ± 2.6
Cu	total content	2.04 ± 0.06	12.7 ± 0.3	8.63 ± 0.16	16.9 ± 0.7	2.11 ± 0.09	9.68 ± 0.07	4.91 ± 0.16	10.7 ± 0.4	0.65 ± 0.03	2.18 ± 0.01	6.66 ± 0.42	5.59 ± 0.17
	dialyzable fraction	0.32 ± 0.01	1.81 ± 0.05	2.21 ± 0.10	0.42 ± 0.01	0.38 ± 0.01	1.37 ± 0.15	0.63 ± 0.04	0.28 ± 0.02	0.32 ± 0.01	1.07 ± 0.01	0.80 ± 0.02	0.81 ± 0.02
	nondialyzable fraction	1.83 ± 0.15	10.6 ± 0.6	6.53 ± 0.38	14.9 ± 0.3	1.68 ± 0.15	8.92 ± 0.27	3.73 ± 0.12	8.92 ± 0.13	0.38 ± 0.02	1.28 ± 0.13	5.14 ± 0.11	4.40 ± 0.14
	sum (dialyzable + nondialyzable)	2.15 ± 0.15	12.4 ± 0.5	8.75 ± 0.38	15.4 ± 0.3	2.07 ± 0.15	10.3 ± 0.1	4.36 ± 0.06	9.20 ± 0.07	0.70 ± 0.02	2.35 ± 0.09	5.94 ± 0.06	5.21 ± 0.12
	agreement, %	106 ± 8	98.2 ± 3.2	101 ± 5	91.1 ± 5.4	97.9 ± 9.4	106 ± 1	89.0 ± 2.1	86.0 ± 2.7	109 ± 7	108 ± 4	89.2 ± 4.9	93.3 ± 4.1
Zn	total content	11.4 ± 0.8	91.6 ± 4.7	19.1 ± 0.5	40.7 ± 0.7	10.0 ± 0.7	42.2 ± 1.6	30.3 ± 0.6	45.4 ± 1.4	12.4 ± 1.1	43.8 ± 0.2	24.6 ± 0.5	17.4 ± 0.5
	dialyzable fraction	0.46 ± 0.07	9.83 ± 0.60	5.32 ± 0.33	1.03 ± 0.04	2.13 ± 0.03	10.7 ± 0.7	5.59 ± 0.37	1.41 ± 0.14	1.87 ± 0.10	5.34 ± 0.64	2.87 ± 0.13	2.13 ± 0.08
	nondialyzable fraction	10.0 ± 0.6	74.4 ± 1.6	12.4 ± 0.2	37.5 ± 0.8	6.55 ± 0.21	35.9 ± 0.6	21.2 ± 1.0	39.2 ± 3.2	10.8 ± 0.7	36.1 ± 2.5	19.2 ± 0.9	14.6 ± 0.2
	sum (dialyzable + nondialyzable)	10.5 ± 0.5	84.2 ± 1.6	17.8 ± 0.1	38.5 ± 0.8	8.68 ± 0.25	46.6 ± 0.9	26.8 ± 0.9	40.6 ± 2.4	12.6 ± 0.6	41.4 ± 2.9	22.1 ± 0.8	16.7 ± 0.1
	agreement, %	91.8 ± 0.1	92.0 ± 5.1	93.2 ± 3.0	94.6 ± 2.9	87.3 ± 6.5	110 ± 5	88.3 ± 2.0	89.4 ± 4.3	103 ± 13	94.7 ± 6.8	89.8 ± 1.8	95.9 ± 2.6

		element content ±SD, ng g^–1^, *n* = 3											
Co	total content	18.2 ± 0.6	58.9 ± 21.8	1788 ± 132	588 ± 35	<LOQ	82.4 ± 3.0	72.3 ± 10.9	189 ± 13	<LOQ	53.0 ± 1.0	395 ± 10	154 ± 2
	dialyzable fraction	3.39 ± 0.11	13.4 ± 3.3	626 ± 27	93.5 ± 7.8	<LOQ	3.28 ± 0.20	17.1 ± 1.5	25.4 ± 0.6	<LOQ	9.60 ± 0.14	25.1 ± 3.5	13.0 ± 1.1
	nondialyzable fraction	13.0 ± 1.1	53.4 ± 18.2	1130 ± 100	450 ± 4	<LOQ	92.6 ± 3.3	59.2 ± 2.4	143 ± 6	<LOQ	37.1 ± 1.1	297 ± 24	119 ± 4
	sum (dialyzable + nondialyzable)	16.4 ± 0.9	66.9 ± 15.2	1756 ± 34	543 ± 6		95.9 ± 2.4	76.4 ± 2.7	169 ± 4		46.7 ± 0.7	322 ± 48	132 ± 5
	agreement, %	90.0 ± 3.3	114 ± 4	98.2 ± 8.8	92.4 ± 6.5		115 ± 7	106 ± 11	89.2 ± 9.3		88.1 ± 2.6	81.8 ± 8.6	85.8 ± 2.3
Ni	total content	706 ± 120	1707 ± 76	1839 ± 11	2471 ± 226	761 ± 17	1161 ± 53	686 ± 137	2381 ± 38	358 ± 26	718 ± 63	1434 ± 313	601 ± 15
	dialyzable fraction	39.7 ± 5.9	264 ± 14	199 ± 20	162 ± 8	<LOQ	201 ± 15	81.7 ± 5.5	200 ± 15	32.5 ± 3.4	197 ± 2	68.9 ± 2.1	105 ± 4
	nondialyzable fraction	578 ± 45	1463 ± 150	1448 ± 25	2138 ± 36	258 ± 8	909 ± 3	448 ± 19	1708 ± 7	290 ± 3	689 ± 18	1187 ± 8	529 ± 19
	sum (dialyzable + nondialyzable)	629 ± 35	1728 ± 96	1647 ± 23	2300 ± 34		1153 ± 76	532 ± 7	1908 ± 20	322 ± 1	885 ± 14	1256 ± 4	628 ± 23
	agreement, %	89.8 ± 9.8	101 ± 2	89.5 ± 0.9	93.3 ± 5.4		99.3 ± 6.9	81.7 ± 6.7	80.1 ± 0.1	90.2 ± 4.3	118 ± 3	87.5 ± 0.3	104.6 ± 5.4
Pb	total content	<LOD	472 ± 97	564 ± 35	1096 ± 65	<LOD	419 ± 61	<LOD	789 ± 30	<LOD	<LOD	1884 ± 96	506 ± 18
	dialyzable fraction	<LOD	35.6 ± 4.9	111 ± 10	36.9 ± 7.8	<LOD	35.1 ± 3.6	<LOD	32.6 ± 1.9	<LOD	<LOD	142 ± 35	31.0 ± 3.8
	nondialyzable fraction	<LOD	469 ± 87	384 ± 18	992 ± 16	<LOD	409 ± 27	<LOD	688 ± 124	<LOD	<LOD	1435 ± 64	382 ± 9
	sum (dialyzable + nondialyzable)		505 ± 65	495 ± 16	1029 ± 16		445 ± 21		720 ± 1			1577 ± 70	413 ± 9
	agreement, %		107 ± 18	87.6 ± 4.6	93.9 ± 6.4		107 ± 16		91.4 ± 3.6			85.6 ± 1.7	82.6 ± 1.6

a Agreement (%) was calculated as (%) (sum (dialyzable + nondialyzable)/total content) × 100%.

**Figure 2:**
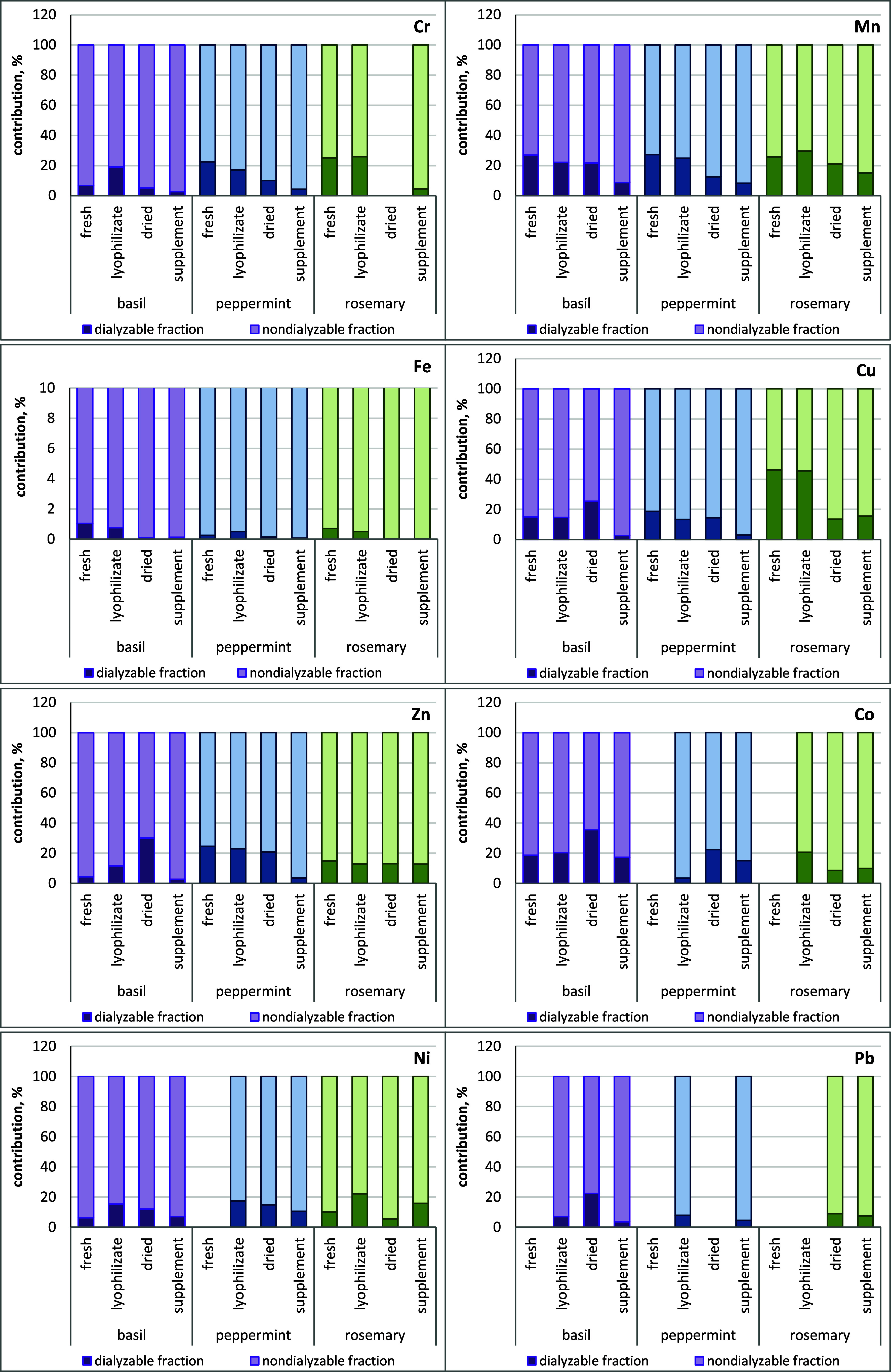
Contribution of elements in the dialyzable and nondialyzable fractions of various forms of basil, peppermint, and rosemary. Figure for Fe has changed maximum scale due to the low content of this element in the dialyzable fraction (<1%).

To assess the ability to release metals from the herbal matrix and their presence in a fraction available for absorption by the intestinal villi, the percentage of the dialyzable fraction of analyte in the sum of metal present in both fractions of plants was calculated. As can be seen in Figure 2, all studied analytes were mainly present in the nondialyzable fraction of herbs obtained by an in vitro digestion method. Comparing the contribution of metals in the dialyzable fraction of tested herbs, we can notice the lowest participation of Fe in each of them (below 1%). In contrast, the contribution of Mn, Cu, Zn, Cr, Co, and Pb was more significant and depended on the form in which a given plant occurred. The lowest metal share in dialyzable fractions was found in plant-based dietary supplements. The highest share of Mn in the dialyzable fraction was observed in fresh and lyophilized basil, mint, and rosemary. In contrast, the share of Cu, Zn and Cr was higher in fresh and lyophilized rosemary and peppermint. Almost all elements were present in higher amounts than in other forms in a dialyzable fraction of dried basil.

### 3.4 Evaluation of Bioaccessibility of Mn, Cu, Zn, Fe, Cr, Co, Ni, and Pb in Herbs

Information about the total content of minerals in food is insufficient to determine its beneficial or harmful effects on human health. For this purpose, it is necessary to assess the bioaccessibility of essential and toxic trace elements. Bioaccessibility depends on many factors related to the element, type of food, efficiency of the digestive process, and absorption capacity in the digestive system.^8,38^ Therefore, using an appropriate research method to understand the interactions between minerals and food components in the gastrointestinal tract is very important. In our work, we used the in vitro gastrointestinal digestion method, which allowed us to determine the fraction of microelements released from the food matrix into the gastrointestinal fluids and available for absorption in the small intestine. The applied in vitro method reflected the digestion of food in the stomach and small intestine, and the use of an artificial dialysis membrane allowed for the simultaneous separation of the dialyzable fraction of elements from the undigested residue of herbs in the intestine. Despite some limitations of this procedure, information about the fraction of minerals that may be available for further use by the human body was obtained relatively quickly.

**Figure 3:**
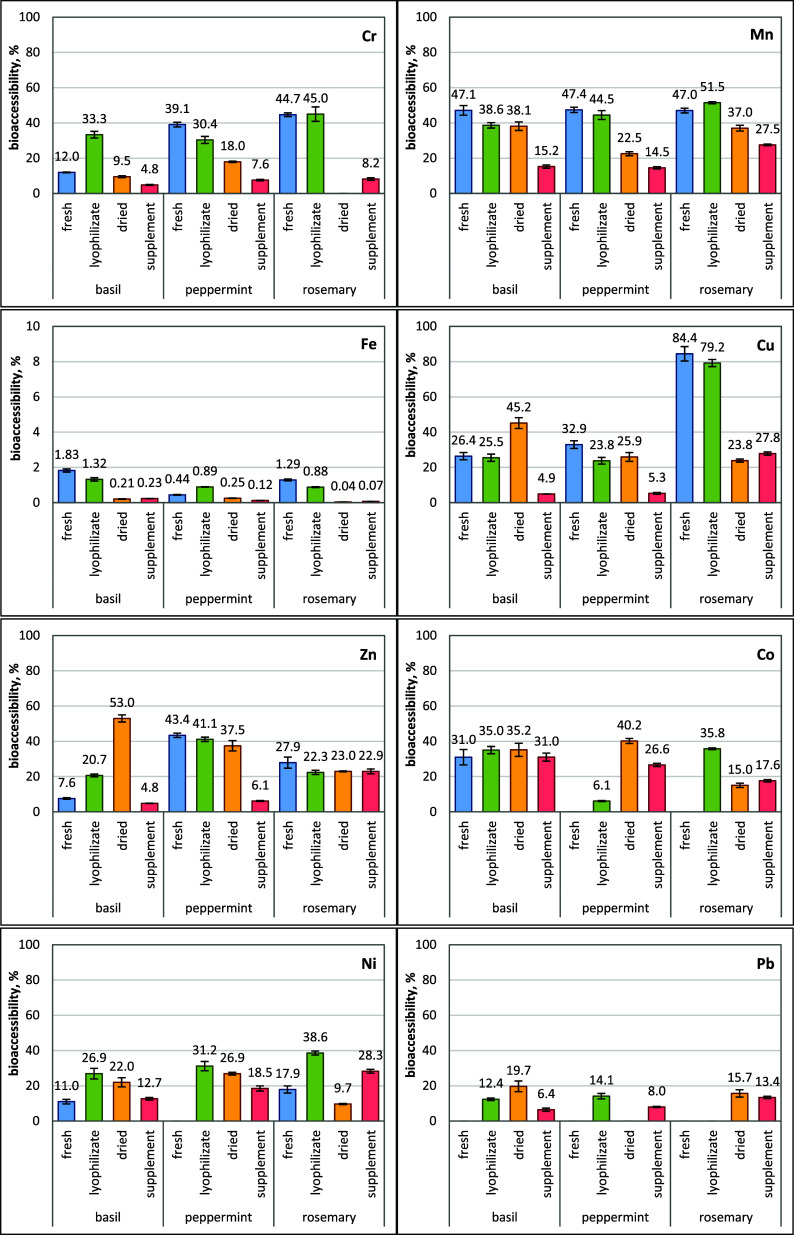
Bioaccessibility of Cr, Mn, Fe, Cu, Zn, Co, Ni, and Pb from basil, peppermint, and rosemary in various forms (fresh, lyophilizate, dried, and dietary supplement) obtained following the in vitro gastrointestinal digestion.

The contents of essential and toxic trace elements in dialyzable and nondialyzable fractions of basil, peppermint, and rosemary after in vitro digestion were determined by the validated ICP–MS method. Based on the obtained results and according to the equation given in Section 2.8, the bioaccessibility of analytes Mn, Cu, Zn, Fe, Cr, Ni, Co, and Pb in herbs in the form of fresh, lyophilized, dried, and dietary supplements was calculated and is presented in Figure 3. Bioaccessibility of Cd, As, and Se was not evaluated due to their low concentration in the dialyzable fraction (below LOD) or low total content in tested herbs (below LOD).

Significant differences were observed in the estimated bioaccessibility values depending on the element, herb, and the form in which it was analyzed. Among all the studied elements, Cu showed the highest bioaccessibility in fresh (84%) and lyophilized (79%) rosemary plants. These values were more than twice as high as those for the same form of basil and peppermint. In other herbs, the bioaccessibility of Cu ranged from 24% to 45%, with low values in dietary supplements containing basil (4.9%) and peppermint (5.3%). The second readily available element was Mn, for which the bioaccessibility value ranged from 37% to 52% in most of the tested plants. Lower values for Mn were found only in all supplements (15–28%) and in dried peppermint (23%). Bioaccessibility of Zn higher than 38% was found in dried (53%) basil, fresh (43%), lyophilized (41%), and dried (38%) peppermint. In the case of all forms of rosemary, such a parameter was at the level of 22–28%. The bioaccessibility of Cr ranged from 12 to 45% in fresh and lyophilized herbs and was below 18% in dried plants and dietary supplements. Good Co bioaccessibility (27–40%) was observed in almost all herbs except lyophilized peppermint (6%), dried rosemary (15%), and its dietary supplement (18%). Among the essential trace elements studied, Fe in all forms of herbs had the lowest bioaccessibility (0.04–1.8%). There may be many reasons for the low bioaccessibility of Fe.^8,39,40^ However, the most important is its occurrence in the form of nonheme Fe in plants, which is much worse absorbed than heme Fe of animal origin. Only Fe in the form of Fe^2+^, together with protons, can be transported through the intestinal mucosa. However, most of the nonheme Fe that enters the gastrointestinal tract occurs in the form of Fe^3+^, which is insoluble and has low bioavailability.^39,40^ Another reason is the presence of substances in plants that bind Fe and limit its absorption or food components, which are released during enzymatic digestion and interact with Fe or other elements.^8,38−41^

Due to the low total content of toxic metals in herbs and their fractions after in vitro digestion, the bioaccessibility of Ni and Pb was evaluated only in this study. Ni showed bioaccessibility in the range of 27–39% in the lyophilized form of all tested herbs and 10–28% in other plant forms. The low values of this parameter (6–16%) were found for Pb in almost all tested herbs, except dried basil (20%). The obtained values may indicate a moderate risk of absorption of these toxic metals in the gastrointestinal tract, which may be unsafe for human health.

In the literature, there is little information about the bioaccessibility of micronutrients in spice plants; therefore, the values obtained were compared to those presented for infusions of slim coffee and herbs or enzymatic extracts of some fruits. The bioaccessibility values of essential trace elements in herbs are similar to the results obtained in infusions of slim coffee (14–33% of Cu, 21–35% of Mn, and 51–66% of Zn)^42^ and herbs as chamomile, peppermint, sage, and nettle (mean values: 16% of Mn and Cu, 22% of Zn, and 2.7% of Fe in linden)^43^ or after in vitro digestion of blackberry, raspberry, blueberry, strawberry (38–43% of Cu, 9–37% of Zn, and 10–52% of Mn),^28^ or goji berry (34% of Mn, 22% of Cu, and 31% of Zn).^44^

**Figure 4:**
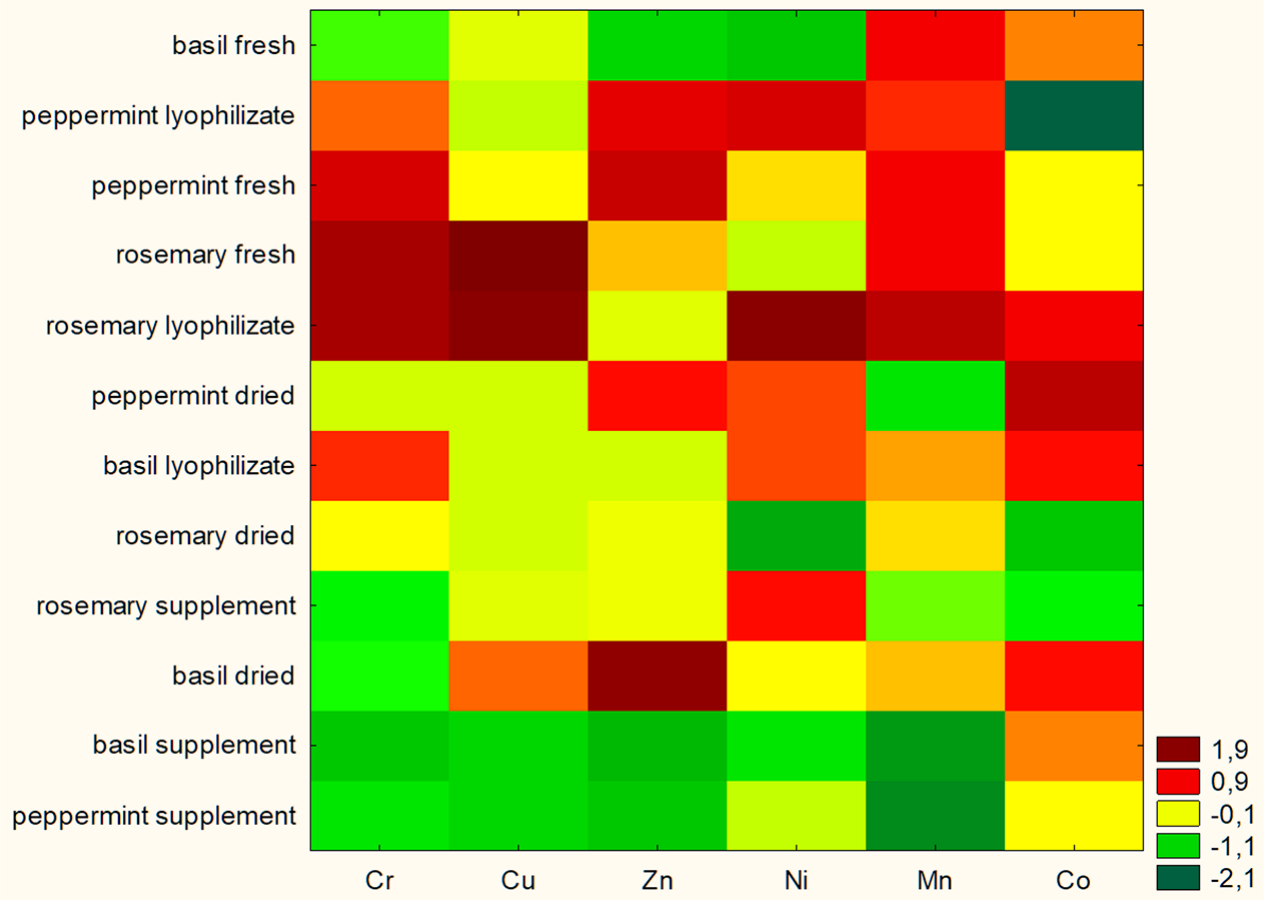
Heatmap of elements’ bioaccessibility from basil, peppermint, and rosemary in various forms (fresh, lyophilizate, dried, and dietary supplement).

In order to visually present the relationships between the bioaccessibility of elements and types of herbs (basil, peppermint, and rosemary) in various forms (fresh, lyophilized, dried, and dietary supplement), the heatmap was prepared and is presented in Figure 4. Elements’ bioaccessibility was displayed, with higher levels depicted by darker red boxes and lower levels shown by darker green boxes. The heatmap revealed that fresh and lyophilized herbs are characterized by the high bioaccessibility of Mn. A similar result was observed for Cr. However, the exception is fresh basil, which has a low bioaccessibility of this element. Additionally, as can be seen from the heatmap, the highest bioaccessibility of elements was observed in lyophilized (Cr, Cu, Ni, Mn, and Co) and fresh (Cr, Cu, and Mn) rosemary, and in lyophilized (Cr, Zn, Ni, and Mn) and fresh (Cr, Zn, and Mn) peppermint. The lowest bioaccessibility of elements was found in all dietary supplements. Only Ni bioaccessibility was high in the rosemary-based dietary supplement. This may be due to the different places of origin of the plants used to produce the supplements or the various technological processes involved in their production.

#### 3.4.1 Influence of Polyphenols on the Bioaccessibility of Essential Trace Elements

The bioaccessibility of elements in herbs may be influenced by components of plant matrices such as dietary fibers, phytic acid, oxalic acid, or polyphenols, called mineral antinutrients.^8,39^ These substances bind minerals in plants in situ, which potentially limits their release during gastrointestinal digestion. This effect mostly depends on the digestibility of the formed chelate (antinutrient–mineral complex) in the gastrointestinal tract. It is also believed that antinutrients originating from other simultaneously ingested food ingredients may form complexes with dietary minerals. Many in vitro and in vivo studies have reported the negative consequences of such substances on Fe, Zn, and other elements’ bioavailability.^39,45−47^ However, it should not be forgotten that these compounds can also possess other health-promoting effects. Polyphenols, represented by flavonoids and tannins, are omnipresent in plant tissues, especially in herbs, where most are conjugated to one or two sugar moieties and form connections with amines, lipids, and organic acids.^8^

The total content of polyphenols in the tested herbs was determined using the Folin–Ciocâlteu method (Table 3) to assess the influence of polyphenols on the availability of essential trace elements. The lowest values of TPC (expressed as gallic acid) were observed in fresh herbs due to the significant contribution of water in their masses used for extraction. Higher values were observed in lyophilized forms of herbs and dietary supplements. The highest amount of polyphenols was found in dried peppermint, basil, and rosemary, originating from organic cultivation. The difference in total polyphenolic content in dietary supplements or lyophilized herbs compared to dried herbs from ecological cultivation may be caused by the effect of various plants’ origin (organic or traditional agriculture) or the way of their preparation for sale (e.g., air drying, high-temperature drying, or irradiation).

Considering the obtained results and Pearson’s correlation, it can be concluded that a higher polyphenol content is associated with a higher total content of elements. Positive Pearson correlation coefficient was obtained in the case of basil and rosemary for Cr, Mn, Fe, Co, Ni, Cu, Zn, and As and for Cr, Mn, Fe, Cu, Zn, and As in peppermint (Table 5). In contrast, a negative correlation was obtained for Cd and Pb in basil and Co and Cd in peppermint. Such observations are consistent with reports of Anjitha et al.^48^ that the production and accumulation of polyphenolic compounds in plants significantly increase during stress induced by the presence of higher concentrations of metal as a result of plant adaptation to harsh growth conditions. On the other hand, polyphenols can exhibit metal-chelating properties due to the presence of hydroxyphenyl and carboxyl groups. It may lead to the reduction of mineral bioaccessibility due to the interaction of polyphenols with metal cations, especially trivalent metals. However, this effect mostly depends on the digestibility of the formed chelate (antinutrient–mineral complex) in the gastrointestinal tract.^39^ In our studies, a higher polyphenol amount correlated positively with the bioaccessibility of Co, Ni, Cu, Zn, and Pb in basil and Ni in peppermint. The negative correlation appeared in the case of Fe in basil, Cr and Mn in peppermint, and Cr, Mn, Fe, Co, Cu, and Zn in rosemary (Table 5). A reduction of absorption of Fe from the human diet is connected with its avid binding with flavonoids, tannins, and phytic acid in plants and the low digestibility of such complexes taking place during human digestion in the gastrointestinal lumen, which was demonstrated in several in vivo studies.^39−41^ It was found that after intestinal absorption of such complexes and during the metabolism of polyphenols, most of them are quickly eliminated in urine and bile without releasing the bound minerals. Zn, in contrast to Fe, has a lower affinity for polyphenols. However, there are only a few in vitro and in vivo studies that examined the effect of polyphenolic-rich food on Zn absorption, and the obtained results are not inconclusive.^49,50^

**Table 5:** Obtained Pearson Correlation Coefficients for the Evaluation of the Correlation Between: TPC and Total Content of Elements, TPC and Elements’ Bioaccessibility, and Total Content of Elements and Elements’ Bioaccessibility

plant	direction	Pearson correlation coefficient (r)		
		TPC—total content of element	TPC—bioaccessibility	total content of element—bioaccessibility
basil	positive	Cr (0.32), Mn (0.54), Fe (0.46), Co (0.71), Ni (0.60), Cu (0.47), Zn (0.35), As (0.76)	Co (0.86), Ni (0.79), Cu (0.46), Zn (0.80), Pb (0.94)	Co (0.46)
	negative	Cd (−0.57), Pb (−0.79)	Fe (−0.68)	Cr (−0.60), Fe (−0.94), Cu (−0.56), Pb (−0.52)
peppermint	positive	Cr (0.33), Mn (0.37), Fe (0.42), Cu (0.47), Zn (0.66), As (0.59)	Ni (0.88)	
	negative	Co (−1.00), Cd (−1.00)	Cr (−0.41), Mn (−0.33)	Cr (−0.91), Mn (−0.47), Fe (−0.33), Ni (−0.82), Cu (−0.83), Zn (−0.61)
rosemary	positive	Cr (0.68), Mn (0.50), Fe (0.84), Co (0.96), Ni (0.75), Cu (0.82), Zn (0.53), As (0.81)		Mn (0.53)
	negative		Cr (−0.50), Mn (−0.41), Fe (−0.83), Co (−0.61), Cu (−0.70), Zn (−0.96)	Cr (−0.55), Fe (−0.59), Co (−0.80), Ni (−0.49), Cu (−0.98), Zn (−0.67)

The study of the effect of the total content of trace elements in herbs on their bioaccessibility showed negative results in the Pearson correlation for most elements (Table 5). This indicates that various food components may affect this value, and a high trace element content does not guarantee their good bioaccessibility. Therefore, studies of the bioaccessibility of trace elements using in vitro methods are still needed because they allow for a real assessment of this value in conditions corresponding to the human body.

#### 3.4.2 Effect of Lyophilization on the Bioaccessibility of Essential Trace Elements

To extend the shelf life of some food products, especially perishable fruits and vegetables, various technological processes are used, such as drying, freezing, or blanching. In recent years, lyophilization has become more and more popular, which involves removing water under reduced temperature and pressure through sublimation. Its advantages include maintaining the original shape, structural integrity, nutritional values, and preservation of aromas. Although this is an expensive process, nowadays, in addition to fruits, you can also find lyophilized herbs on the market that retain their healthy properties and good flavor.

In our work, some of the fresh herb samples were lyophilized to extend their shelf life. Then, fresh and lyophilized herbs were subjected to an in vitro digestion procedure to assess the impact of this method of herb processing on the bioaccessibility of essential trace elements. For elements with high bioaccessibility, such as Mn, Cu, and Zn, the obtained values were compared and subjected to statistical tests to confirm or exclude differences. Statistically significant differences between standard deviations (SD) of bioaccessibility of elements in plants were verified using the one-tailed Fisher-Snedecor F test. The critical parameter for this test (*F*_crit_) was 19.00 at the 95% significance level (α = 0.05).^51^ The calculated values of the *F* parameter (*F*_calc_) were lower than the value of *F*_crit_ (*F*_calc_ < *F*_crit_) (Table S3), which allowed it to be concluded that the SD of the compared metal bioaccessibility results did not differ statistically significantly. Therefore, the two-sided Student’s *t*-test was used to compare the mean bioaccessibility of the elements. The critical value (*t*_crit_) was set at 2.776 (α = 0.05)^51^ and compared with the calculated values (*t*_calc_). Good agreement of bioaccessibility in fresh and lyophilized herbs (*t*_calc_ < *t*_crit_) was obtained for Mn, Cu, and Zn in rosemary; Mn and Zn in peppermint; and Cu in basil (Table S3). Considering the previously estimated expanded uncertainty values of measurements (Table 2), the ranges of bioaccessibility values of Mn, Cu, and Zn in fresh and lyophilized herbs were calculated. An overlap of these ranges was observed for Mn, Cu, and Zn in rosemary, Mn and Zn in peppermint, and Cu in basil, which shows good agreement between these results (Table S3). This may indicate that the lyophilization process does not significantly affect the bioaccessibility of these elements. In both comparisons, good agreement of results for Mn, Cu, and Zn was observed for rosemary, the herb with the lowest water content among tested plants (74% vs 90%). Therefore, further studies should be conducted on the influence of the lyophilization process on the bioaccessibility of essential trace elements in herbs with different water content.

This study revealed that the bioaccessibility of elements varied in tested spice plants depending on the type of plant, its form, and origin. The comparison of various forms of herbs shows that the bioaccessibility of trace elements is the highest in fresh and lyophilized herbs and the lowest in dietary supplements. It may be connected with the origin of spice plants as fresh, dried, and dietary supplements came from different sources. The determined phenolic content mostly positively correlates with the total element content in herbs, but their effect on the element bioaccessibility is unclear. Therefore, further research is needed to understand the exact factors influencing the bioaccessibility of elements in plants. The results obtained in our work may be a guide for consumers to use fresh or lyophilized herbs more often in their diet.

## Supporting Information Available

Risk exposure assessment considering the consumption of 1 g of spice plants daily; RDA assessment; considering the Mn, Fe, Cu, and Zn mean levels and the daily consumption of 1 g of spice plant daily; and calculated values of F- and *t* tests for comparisons of Mn, Cu, and Zn bioaccessibility in fresh plants with bioaccessibility of these elements in plants lyophilizate obtained with in vitro digestion model (PDF)
