# Supplementary material for: Evaluation of the Bioaccessibility of Essential and Toxic Trace Elements in Basil, Peppermint, and Rosemary Using an In Vitro Gastrointestinal Digestion Model
Source: J Agric Food Chem. 2025 Feb 28;73(10):6189–204. doi: 10.1021/acs.jafc.4c10940 (PMC11907404; doi:10.1021/acs.jafc.4c10940)
Supplement: Supplementary file 1 — jf4c10940_si_001.pdf [file jf4c10940_si_001.pdf]

Supplementary materials

Evaluation of the bioaccessibility of essential and toxic trace elements in basil, peppermint and rosemary using an *in vitro* gastrointestinal digestion model

Sylwia Sajkowska[a,b], Justyna Moskwa[c], Katarzyna Socha[c], Barbara Leśniewska[b,*]

[a] *Doctoral School of the University of Bialystok, Ciołkowskiego 1K, Bialystok 15-245, Poland*

[b] *Department of Analytical and Inorganic Chemistry, Faculty of Chemistry, University of Bialystok, Ciołkowskiego 1K, Bialystok 15-245, Poland*

[c] *Department of Bromatology, Faculty of Pharmacy with Division of Laboratory Medicine, Medical University of Bialystok, Mickiewicza 2D, Bialystok 15-222, Poland*

Table S1. Risk exposure assessment considering the consumption of 1 gram of spice plants daily.

Spice plant	Element			
	Cd	As	Pb	
	MI, µg kg^{-1} b.w. (% PTMI)	% BMDL (0.06 µg kg^{-1} b.w.)	% BMDL (0.63 µg kg^{-1} b.w.)	% BMDL (1.5 µg kg^{-1} b.w.)
basil fresh	-	1.07	-	
basil lyophilizate	0.01 (0.03)	3.89	1.25	0.52
basil dried	0.01 (0.06)	31.9	1.49	0.63
basil supplement	0.03 (0.10)	10.4	2.90	1.22
peppermint fresh	-	0.56	-	-
peppermint lyophilizate	0.02 (0.09)	5.11	1.11	0.47
peppermint dried	0.02 (0.06)	1.85	-	-
peppermint supplement	0.06 (0.23)	3.75	2.09	0.88
rosemary fresh	-	0.75	-	-
rosemary lyophilizate	0.01 (0.04)	2.72	-	-
rosemary dried	0.01 (0.04)	7.61	4.98	2.09
rosemary supplement	0.004 (0.02)	3.78	1.34	0.56

MI – Monthly Intake
b.w. – reference body weight (60 kg)
PTMI – Temporary Tolerable Monthly Intake: Cd 25 µg kg^{-1} b.w.
BMDL – Benchmark Dose Lower confidence limit: As 0.06 µg kg^{-1} b.w. for skin cancer, Pb 0.63 µg kg^{-1} b.w. and 1.5 µg kg^{-1} b.w. for the kidney and cardiovascular effects, respectively

Table S2. Recommended Dietary Allowance (RDA) assessment; considering the Mn, Fe, Cu and Zn mean levels and the daily consumption of 1 gram of spice plant daily. In brackets are given values for consumption of 25 g of fresh plant, e.g. as a pesto.

Spice plant	Consumption	Element			
		Mn	Fe	Cu	Zn
basil fresh	Intake, µg	46.7 (1168)	33.6 (841)	2.04 (50.9)	11.4 (285)
	% RDA (women)	2.59 (64.9)	0.19 (4.67)	0.23 (5.65)	0.14 (3.57)
	% RDA (men)	2.03 (50.8)	0.34 (8.41)	0.23 (5.65)	0.10 (2.59)
basil lyophilizate	Intake, µg	345	319	12.7	91.6
	% RDA (women)	19.1	1.77	1.41	1.15
	% RDA (men)	15.0	3.19	1.41	0.83
basil dried	Intake, µg	139	769	8.63	19.1
	% RDA (women)	7.72	4.27	0.96	0.24
	% RDA (men)	6.04	7.69	0.96	0.17
basil supplement	Intake, µg	53.3	1187	16.9	40.7
	% RDA (women)	2.96	6.59	1.88	0.51
	% RDA (men)	2.32	11.9	1.88	0.37
peppermint fresh	Intake, µg	15.7 (392)	44.9 (1122)	2.11 (52.8)	9.97 (249)
	% RDA (women)	0.87 (21.8)	0.25 (6.2)	0.23 (5.9)	0.12 (3.1)
	% RDA (men)	0.68 (17.1)	0.45 (11.2)	0.23 (5.9)	0.09 (2.3)
peppermint lyophilizate	Intake, µg	105	257	9.68	42.2
	% RDA (women)	5.83	1.43	1.08	0.53
	% RDA (men)	4.56	2.57	1.08	0.38
peppermint dried	Intake, µg	42.4	222	4.91	30.3
	% RDA (women)	2.36	1.24	0.55	0.38
	% RDA (men)	1.84	2.22	0.55	0.28
peppermint supplement	Intake, µg	132	449	10.7	45.4
	% RDA (women)	7.33	2.49	1.19	0.57
	% RDA (men)	5.74	4.49	1.19	0.41
rosemary fresh	Intake, µg	15.3 (381)	128 (3208)	0.65 (16.1)	12.4 (310)
	% RDA (women)	0.85 (21.2)	0.71 (17.8)	0.07 (1.8)	0.16 (3.9)
	% RDA (men)	0.66 (16.6)	1.28 (32.1)	0.07 (1.8)	0.11 (2.8)
rosemary lyophilizate	Intake, µg	81.4	636	2.18	43.8
	% RDA (women)	4.52	3.53	0.24	0.55
	% RDA (men)	3.54	6.36	0.24	0.40
rosemary dried	Intake, µg	38.1	897	6.66	24.6
	% RDA (women)	2.11	4.98	0.74	0.31
	% RDA (men)	1.65	8.97	0.74	0.22
rosemary supplement	Intake, µg	24.5	352	5.59	17.4
	% RDA (women)	1.36	1.95	0.62	0.22
	% RDA (men)	1.07	3.52	0.62	0.16

levels of RDA for women and men, respectively: Mn 1.8 mg and 2.3 mg, Fe 18 mg and 10 mg, Cu 0.9 mg and 0.9 mg, Zn 8 mg and 11 mg

Table S3. Calculated values of F- and t-tests for comparisons of Mn, Cu and Zn bioaccessibility in fresh plants with bioaccessibility of these elements in plants lyophilizate obtained with *in vitro* digestion model. Significant differences are italicized.

element	F_{calc}			t_{calc}		
	**basil**	**peppermint**	**rosemary**	**basil**	**peppermint**	**rosemary**
**Mn**	3.094	2.823	0.265	*4.637*	1.740	2.634
**Cu**	1.015	1.298	4.072	0.528	*5.517*	1.985
**Zn**	2.916	1.050	4.267	*25.13*	2.268	2.762

Bioaccessibility ± U, %, *k* = 2

element	**basil**		**peppermint**		**rosemary**	
	**fresh**	**lyophilizate**	**fresh**	**lyophilizate**	**fresh**	**lyophilizate**
**Mn**	47.1 ± 4.0	38.6 ± 3.3	47.4 ± 4.1	44.5 ± 3.8	47.0 ± 4.0	51.5 ± 4.4
**Cu**	26.4 ± 2.6	25.5 ± 2.5	32.9 ± 3.2	23.8 ± 2.3	84.4 ± 8.3	79.2 ± 7.8
**Zn**	7.56 ± 0.83	20.7 ± 2.3	43.4 ± 4.8	41.1 ± 4.5	27.9 ± 3.1	22.3 ± 2.5

F_{crit} = 19.00 (f=4, α = 0.05)

t_{crit} =2.776 (f=4, α = 0.05)
